# The oncogenic kinase TOPK upregulates in psoriatic keratinocytes and contributes to psoriasis progression by regulating neutrophils infiltration

**DOI:** 10.1186/s12964-024-01758-9

**Published:** 2024-08-01

**Authors:** Fanfan Zeng, Shuaixian Du, Mengjun Wu, Chan Dai, Jianyu Li, Jinbiao Wang, Guoyun Hu, Pengcheng Cai, Lin Wang

**Affiliations:** 1 Department of Clinical Laboratory, Union Hospital, Tongji Medical College, Huazhong University of Science and Technology, Wuhan, Hubei China; 2 Department of Otorhinolaryngology, Union Hospital, Tongji Medical College, Huazhong University of Science and Technology, Wuhan, Hubei China; 3 Department of Immunology, School of Basic Medicine, Tongji Medical College, Huazhong University of Science and Technology, Wuhan, Hubei China

**Keywords:** Psoriasis, Keratinocytes, Neutrophils, TOPK, Targeted therapy

## Abstract

**Background:**

T-LAK cell-oriented protein kinase (TOPK) strongly promotes the malignant proliferation of cancer cells and is recognized as a promising biomarker of tumor progression. Psoriasis is a common inflammatory skin disease featured by excessive proliferation of keratinocytes. Although we have previously reported that topically inhibiting TOPK suppressed psoriatic manifestations in psoriasis-like model mice, the exact role of TOPK in psoriatic inflammation and the underlying mechanism remains elusive.

**Methods:**

GEO datasets were analyzed to investigate the association of TOPK with psoriasis. Skin immunohistochemical (IHC) staining was performed to clarify the major cells expressing TOPK. TOPK conditional knockout (cko) mice were used to investigate the role of TOPK-specific deletion in IMQ-induced psoriasis-like dermatitis in mice. Flow cytometry was used to analyze the alteration of psoriasis-related immune cells in the lesional skin. Next, the M5-induced psoriasis cell model was used to identify the potential mechanism by RNA-seq, RT-RCR, and western blotting. Finally, the neutrophil-neutralizing antibody was used to confirm the relationship between TOPK and neutrophils in psoriasis-like dermatitis in mice.

**Results:**

We found that TOPK levels were strongly associated with the progression of psoriasis. TOPK was predominantly increased in the epidermal keratinocytes of psoriatic lesions, and conditional knockout of TOPK in keratinocytes suppressed neutrophils infiltration and attenuated psoriatic inflammation. Neutrophils deletion by neutralizing antibody greatly diminished the suppressive effect of TOPK cko in psoriasis-like dermatitis in mice. In addition, topical application of TOPK inhibitor OTS514 effectively attenuated already-established psoriasis-like dermatitis in mice. Mechanismly, RNA-seq revealed that TOPK regulated the expression of some genes in the IL-17 signaling pathway, such as neutrophils chemokines CXCL1, CXCL2, and CXCL8. TOPK modulated the expression of neutrophils chemokines via activating transcription factors STAT3 and NF-κB p65 in keratinocytes, thereby promoting neutrophils infiltration and psoriasis progression.

**Conclusions:**

This study identified a crucial role of TOPK in psoriasis by regulating neutrophils infiltration, providing new insights into the pathogenesis of psoriasis.

**Supplementary Information:**

The online version contains supplementary material available at 10.1186/s12964-024-01758-9.

## Background

Psoriasis is one of the most common immune-mediated chronic inflammatory skin disorders occurring equally among males and females [1]. There are over 60 million patients, including children and adults, suffering from psoriasis worldwide [2, 3]. Psoriasis increases the risk of cardiovascular disease [4], non-alcoholic fatty liver disease [5], metabolic syndrome [5], respiratory disorder [6, 7], psychiatric disorders [8, 9], and autoimmune inflammatory diseases [10]. Psoriasis not only seriously affects the physical and mental health of patients, but also poses a serious threat to global health issues.

Epidermal keratinocytes and neutrophils play crucial roles in psoriasis pathogenesis and progression. Keratinocytes are the main constituent cells of the epidermis and play important roles in different phases of the skin immune response [11]. In response to external stimuli, keratinocytes release a number of cytokines and antimicrobial peptides, for example, LL-37. LL-37 binds to self-nucleic acid to form complexes that act as antigens to initiate psoriasis pathogenesis [12]. Apart from keratinocytes, neutrophils have also been reported to produce antimicrobial peptides (AMPs), thus contributing to psoriasis pathogenesis. In psoriatic lesions, inflammatory cytokines act on keratinocytes and activate transcription factors, such as NF-κB and STAT3, to induce the expression of chemokines for neutrophils [13–15]. Infiltrated neutrophils accelerate skin inflammation and pathological changes by releasing multiple psoriasis-related proinflammatory cytokines and aggregating on the stratum corneum of the epidermis to form Munro’s microabscesses. In addition, infiltrated neutrophils could produce some inflammatory mediators, such as protease 3 and LCN2, which promote keratinocytes hyperproliferation and psoriasis progression [16, 17]. However, the interplay between epidermal keratinocytes and infiltrated neutrophils in psoriasis, and the potential regulatory factors between keratinocytes and neutrophils have not been fully addressed.

TOPK is an oncogenic serine-threonine kinase belonging to the mitogen-activated protein kinase kinase (MAPKK) family [18]. TOPK is not or is lowly expressed in normal cells, whereas it is abundantly expressed in cells with high proliferative capacity (e.g., malignant tumor cells) [19]. TOPK is upregulated in various cancers and contributes to cancer cell transformation, malignant proliferation, and cancer metastasis [19]. TOPK is reported to be highly correlated with tumor progression and disease prognosis [20]. TOPK is increasingly recognized as a potential and promising target for cancer treatment [21, 22]. In addition to its role in malignant cancers, recent studies have reported that TOPK is also associated with inflammatory skin disease. Fan et al. showed that TOPK promoted solar UV-induced skin dermatitis [23]. Our previous study identified that targeting TOPK markedly repressed solar UV-induced skin dermatitis [24]. Our recent study and Lu et al. found that topical application of TOPK inhibitor suppressed pathologic changes in psoriasis-like dermatitis in mice, implying that TOPK may be involved in psoriatic inflammation [25, 26]. However, the detailed mechanism underlying TOPK and psoriasis progression remains largely unknown.

By using GEO datasets, psoriasis patients’ samples, conditional knockout mice, in vivo psoriatic mice model, and in vitro psoriatic cell model, we determined that TOPK increased in psoriatic keratinocytes and TOPK levels in lesions were positively related to the progression of psoriasis. Increased TOPK in keratinocytes promoted the progression of psoriasis by regulating neutrophils infiltration. This study identifies that in addition to its well-known oncogenic role, keratinocyte-expressed TOPK is involved in the inflammatory skin disease psoriasis, providing new insights into the pathogenesis of psoriasis.

## Materials and methods

### Clinical samples

Healthy skin specimens were derived from the surgical waste of patients in Wuhan Union Hospital (Wuhan, China). Psoriasis skin specimens were derived from paraffin-embedded biopsy in the Clinical Specimen Repository of Wuhan Union Hospital. Written informed consent from donors was obtained. The human experiments were performed strictly following the tenets of the Helsinki Declaration, and this study was approved by the Medical Ethics Committee of Wuhan Union Hospital, Tongji Medical College, Huazhong University of Science and Technology (HUST) (UHCT230205).

### Mice

All mice used in this study were C57BL/6 background. The WT mice were ordered from Huafukang Biotechnology Co., Ltd. (Beijing, China). The TOPK-flox mice and K14-Cre ERT were ordered from Cyagen Biotechnology Co., Ltd. (Suzhou, China; https://www.cyagen.com/cn/zh-cn/). All mice were bred and maintained at the Animal Centre of Tongji Medical College, HUST, under specific pathogen-free conditions. To induce TOPK specific deletion in keratinocytes, K14-CreERT-TOPK-flox/flox mice were injected intraperitoneally with 75 mg/kg tamoxifen (Selleck, cat: S1238, dissolved in corn oil) for five consecutive days, and control mice were injected intraperitoneally with an equal volume of corn oil. The mice were then kept normally for two more weeks. The successful deletion of TOPK was verified by IHC staining (supplementary material 1) before being used for the following experiments. All mice were killed using 5% isoflurane, followed by decapitation. All mouse experiments were performed following the Guidelines for the Care and Use of Laboratory Animals of HUST and approved by the Laboratory Animal Ethics Committee of Tongji Medical College, HUST.

### Cell culture

HaCaT cells were a human keratinocytes cell line ordered from the American Type Culture Collection. HaCaT cells were cultured with DMEM containing 10% FBS under 37 °C and 5% CO2 in a cell incubator.

### Induction of psoriatic cell model

The psoriatic cell model was induced as previously reported model [27, 28]. HaCaT cells were cultured and treated with five cytokines (named M5), including IL-22, IL-17A, IL-1a, TNF-α, and oncostatin M to induce psoriatic cell model. The concentration of IL-22 (PeproTech, cat: 200-22), IL-17A (PeproTech, cat: 200-17), TNF-α (PeproTech, cat: 300-01A), IL-1a (PeproTech, cat: 200-01A), and oncostatin M (PeproTech, cat: 300-10H) used in this study was 10 ng/mL. Consistently with Guilloteau et al.‘s study, M5 cocktail cytokines induced psoriasis-like inflammation in HaCaT cells in vitro in our study (supplementary material 2).

### Induction of psoriatic mice model

Psoriasis-like dermatitis was induced using 7 to 8-week-old mice. The skin on the back was shaved one day before the model induction. Mice were treated topically by 5% imiquimod (IMQ) cream (Sichuan Med-Shine Pharmaceutical Co., Ltd., China). The single dose for dorsal skin is 62.5 mg, and for ear skin is 10.0 mg. The single dose of OTS514 cream was 100 mg for the dorsal skin and 20 mg for the ear skin.

### Western blotting

Cells were cultured and treated in 12-well cell plates. After treatment, lysis buffer was added directly into the cell culture plate. HaCaT cells were transferred into 1.5mL EP tubes and lysed on ice for 10 min. Afterwards, collect the cell lysis and degenerated after adding protein loading buffer (Beyotime Biotechnology). Ran the samples with 10% SDS-PAGE gels and blotted onto polyvinylidene fluoride membranes (Millipore). The primary antibodies used in this were as follows: STAT3, CST, cat: 9139T; p-STAT3, CST, cat: 9145T; NF-kappaB p65, CST, cat: 8242T; p-NF-kappaB p65, CST, cat: 3033T; β-actin, Proteintech, cat: 66009-1-Ig. All antibodies for western blot experiments were used with a dilution of 1:1000. The concentration of OTS514 used for cell treatment in this assay is 5nM, 10nM or 20nM, as indicated in the figures. Inhibitors JSH-23 (Selleck, cat: S7351) and stattic (Selleck, cat: S7024) used in this study were 30µM and 10µM, respectively.

### RNA sequencing analysis

HaCaT cells were seeded and grown to 70% confluence. The cells were preincubated with 10nM OTS514 for 12 h and then treated together with 10ng/mL M5 cocktail cytokines for 24 h. Total RNA was isolated by using TRIzol reagent (Invitrogen). RNA sequencing was performed by BGI Co., L (Shenzhen, China). The filtered reads were mapped to the reference genome using HISAT2 software (v2.1.0). StringTie (v2.1.5) was used to compare the read count values on each gene as the original expression of the gene, and FPKM was used to standardize the expression. The differential expression of genes was analyzed by DESeq2 (v1.30.1) with screening conditions: P-value < 0.05 and fold change ≥ 2.

### Flow cytometry

Mice skin cells were isolated as previously described [29]. Ear skin was used for flow cytometry analysis. In brief, mice ears were cut into pieces and then incubated with digestion buffer in a shaking incubator at 37 ℃ for 60 min. The suspensions were then filtered through 100 mm cell strainers. The cells were centrifuged and then resuspended in PBS for subsequent cell staining. Finally, the cells were subjected to flow cytometry analysis by using CytoFLEX (Beckman Coulter, USA). The data was analyzed using FlowJo-V10 software. The antibodies using for flow cytometry included BV605-CD45 (Biolegend, cat: 103139), PE-CD3 (Biolegend, cat: 100206), Percp-cy5.5-CD4 (Biolegend, cat: 100434), FITC-γδTCR (Biolegend, cat: 118105), PE-cy7-CD11c (BD Pharmingen, cat: 558079), and APC-Ly6G (Biolegend, cat: 108412). Live cells were labeled by using the Live/dead Cell Stain Kit (Thermo Fisher, cat: L34963).

### Statistics

All statistical analyses were performed by using GraphPad Prism (GraphPad Software, San Diego, USA). Data were representative of two or three independent experiments and were expressed as mean ± SEM. Statistical differences were determined by using unpaired or paired Student’s t-tests, Mann-Whitney U-tests, or one-way ANOVA followed by Dunnett’s multiple comparison test, as appropriate. Statistical methods used for each analysis were described in the figure legends. p values less than 0.05 were considered statistically significant. *P < 0.05, **P < 0.01, ***P < 0.001, ****P < 0.0001.

## Results

### TOPK is increased in psoriatic keratinocytes and TOPK levels are associated with psoriasis progression

**Fig. 1:**
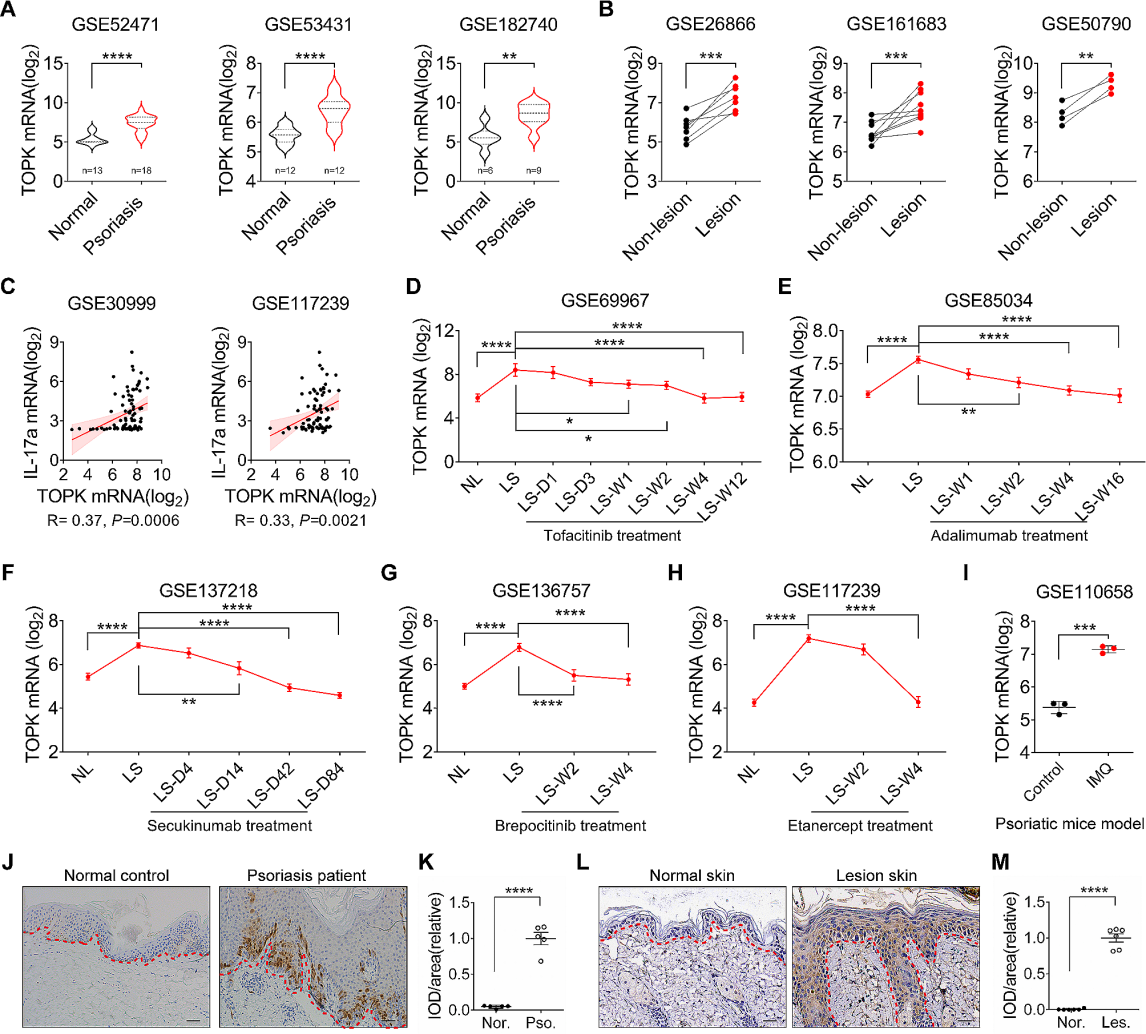
TOPK levels correlate with psoriatic progression, and TOPK is increased in the epidermal keratinocytes of psoriatic lesions. (**A**) GEO datasets analysis of TOPK mRNA in normal skin of non-psoriatic individuals and lesional skin of psoriasis patients. (**B**) GEO datasets analysis of TOPK mRNA in paired non-lesional skin and lesional skin of psoriasis patients. (**C**) Pearson correlation analysis of TOPK mRNA and IL-17 A mRNA in the lesional skin of psoriasis patients. (**D**-**H**) GEO datasets analysis of TOPK mRNA before and after therapy in psoriasis patients. (**I**) GEO datasets analysis of TOPK mRNA in normal skin of control mice and lesional skin of IMQ-induced psoriatic model mice. (**J**) Representative IHC staining images of anti-TOPK antibody in normal skin of non-psoriatic individuals and lesional skin of psoriasis patients. (**K**) Semiquantitative analysis of TOPK relative staining intensity in human epidermal keratinocytes. (**L**) Representative IHC staining images of anti-TOPK antibody in normal mice skin and lesional skin of psoriatic model mice. (**M**) Semiquantitative analysis of TOPK relative staining intensity in mice epidermal keratinocytes. Significance was determined by (A, I, K, and M) Student’s t-test, (B) paired Student’s t-test, (C) Pearson correlation analysis, and (D-H) one-way ANOVA followed by Dunnett’s multiple comparisons test. *P < 0.05, **P < 0.01, ***P < 0.001, ****P < 0.0001. Scale bar = 50 μm. NL, non-lesional skin. LS, lesional skin. D, day. W, week

To determine the expression changes of TOPK in psoriatic lesions, we screened and analyzed GEO datasets of transcriptome profiles from psoriatic patients and non-psoriatic individuals. The data indicated that TOPK was markedly increased in the lesional skin of psoriasis patients relative to the normal skin of non-psoriatic individuals (Fig. 1A). Besides, analysis of GSE datasets containing paired non-lesional and lesional skin of psoriasis patients showed that TOPK was also obviously elevated in the lesional skin relative to the paired non-lesional skin (Fig. 1B). Furthermore, correlation analysis showed that TOPK levels were positively correlated with IL-17 A levels in psoriasis lesional skin (Fig. 1C). Moreover, analyzing GEO datasets of psoriatic pre-treated and post-treated skin samples indicated that before treatment, TOPK levels were higher in the lesional skin than those in the non-lesional skin. However, after 12 weeks of tofacitinib treatment, TOPK levels decreased significantly, coinciding with the attenuation of psoriasis (Fig. 1D). Similarly, after treatment with adalimumab for 16 weeks, TOPK in the lesional skin significantly decreased to near the baseline levels (Fig. 1E). Whether psoriasis patients were treated with secukinumab, brepocitinib, or etanercept, TOPK levels gradually decreased as the illness improved (Fig. 1F-H). Not only for clinical psoriasis, analyzing GEO datasets of mice skin samples indicated that TOPK levels were also significantly elevated in the lesional skin of psoriatic model mice (Fig. 1I). Next, we performed IHC staining to clarify the expression pattern of TOPK in the skin. The results showed that TOPK was primarily expressed in the highly proliferative epidermal keratinocytes of the psoriatic lesions. For human skin, TOPK was not stained in the normal skin, whereas TOPK was strongly stained in the epidermis of psoriasis lesional skin. The staining intensity of TOPK in psoriatic epidermal keratinocytes was significantly increased compared with the normal skin (Fig. 1J and K). Importantly, a similar staining pattern was obtained in mice skin. TOPK was barely stained in normal skin and intensely stained in epidermal keratinocytes of psoriasis-like lesions in mice (Fig. 1L and M). Altogether, these results indicate that TOPK increases in psoriatic keratinocytes and TOPK levels are associated with the psoriasis progression, implying that keratinocytes-expressing TOPK may contribute to the pathogenesis of psoriasis.

### Keratinocytes deletion of TOPK attenuates the progression of psoriasis-like dermatitis in mice

**Fig. 2:**
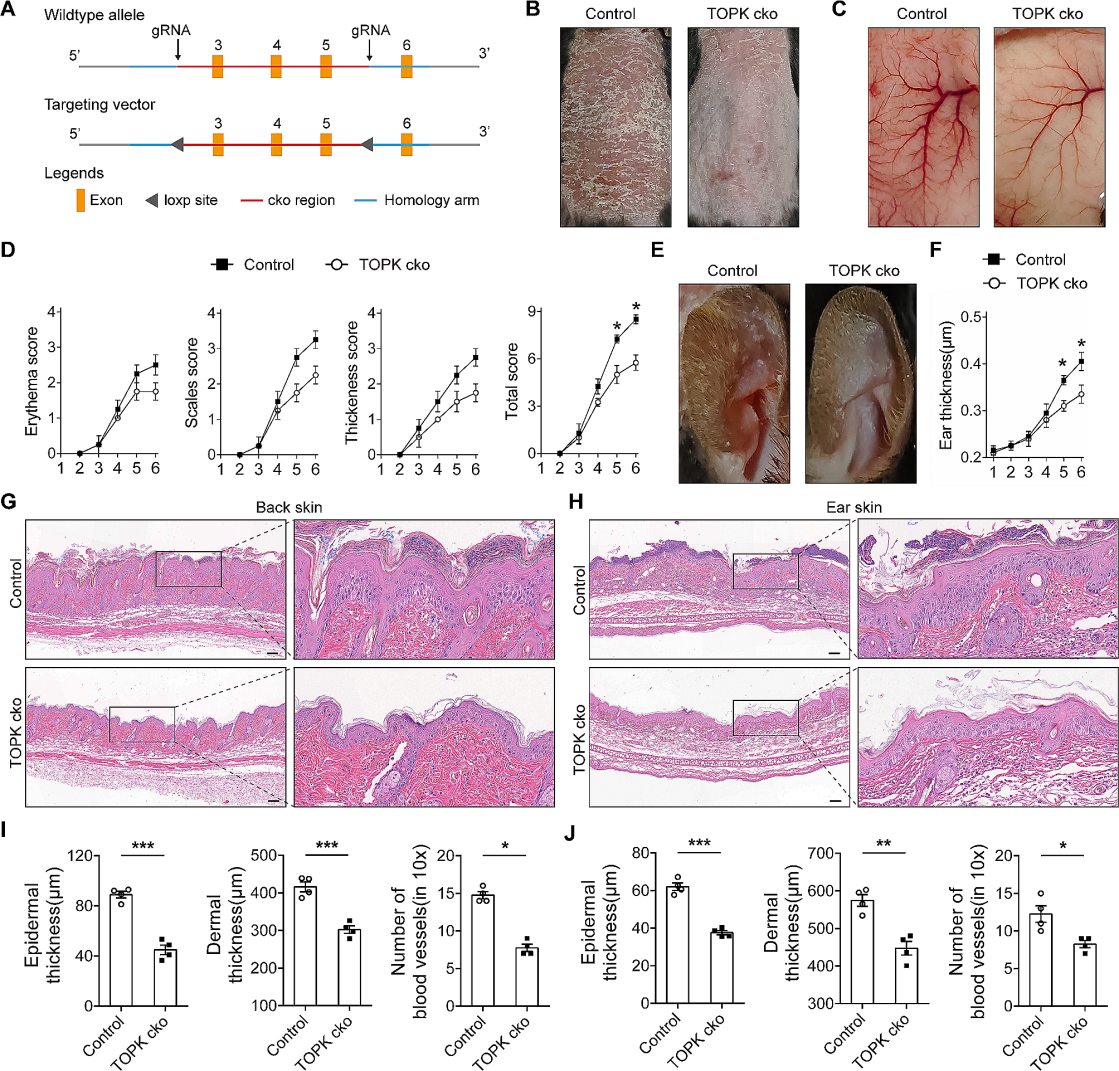
TOPK cko attenuates psoriasis-like dermatitis in IMQ-induced psoriatic model mice. (**A**) Genotyping strategy of TOPK conditional knockout in mice. (**B**) Macroscopic images of psoriasis-like manifestations in control mice and TOPK cko mice after inducing psoriatic model. (**C**) Images of blood vessels within the skin of control psoriatic model mice and TOPK cko psoriatic model mice. (**D**) PASI scoring of control psoriatic model mice and TOPK cko psoriatic model mice. *n* = 4 per group. (**E**) Macroscopic images of psoriasis-like manifestations in ear skin of control psoriatic model mice and TOPK cko psoriatic model mice. (**F**) Statistical analysis of ear thickness measured by vernier caliper. *n* = 4 per group. (**G** and **H**) Representative HE staining images of back skin (**G**) and ear skin (**H**) of control psoriatic model mice and TOPK cko psoriatic model mice. (**I** and **J**) Statistical data of epidermal thickness, dermal thickness and number of blood vessels in back skin (**I**) and ear skin (**J**). Scale bar = 100 μm. Significance was determined by (D) Mann-Whitney U test and (F) Student’s t-test. Significance of Fig. 2I and J was determined by Student’s t-test or Mann-Whitney U test, as appropriate. *P < 0.05, **P < 0.01, ***P < 0.001

To address the role of increased expression of TOPK in psoriasis, TOPK cko mice were established and subjected to inducing psoriasis-like dermatitis (Fig. 2A). The data showed that, relative to WT mice, TOPK cko mice exhibited slighter psoriatic manifestations, including scaling, erythema, and epidermal thickening after inducing psoriasis-like dermatitis (Fig. 2B). Vessel hyperplasia, one of the typical hallmarks of psoriasis, was also obviously relieved in the lesions of TOPK cko psoriasis-like model mice (Fig. 2C). Using the Psoriasis Area Severity Index (PASI) to assess the severity of psoriasis showed that the erythema score, scales score, thickness score, and total score were decreased in TOPK cko psoriasis-like model mice (Fig. 2D). In addition, mice ear skin was also applied to confirm the effect of TOPK cko on the pathogenesis of psoriasis. The data indicated that deletion of TOPK in keratinocytes in the ear skin attenuated psoriasis-like manifestations and ear skin thickening in psoriatic model mice (Fig. 2E and F). Next, mice skin samples were subjected to HE staining to determine the effect of TOPK cko on pathological changes of psoriasis (Fig. 2G and H). The images showed that deletion of TOPK in keratinocytes, whether in back skin or ear skin, attenuated psoriasis-like pathological changes, including epidermal thickening, dermal thickening, and vessel hyperplasia in psoriatic model mice (Fig. 2I and J). Collectively, these data suggest that keratinocytes-expressed TOPK promotes the progression of psoriasis-like dermatitis in mice.

### TOPK deletion in keratinocytes decreases neutrophils infiltration in psoriatic model mice

**Fig. 3:**
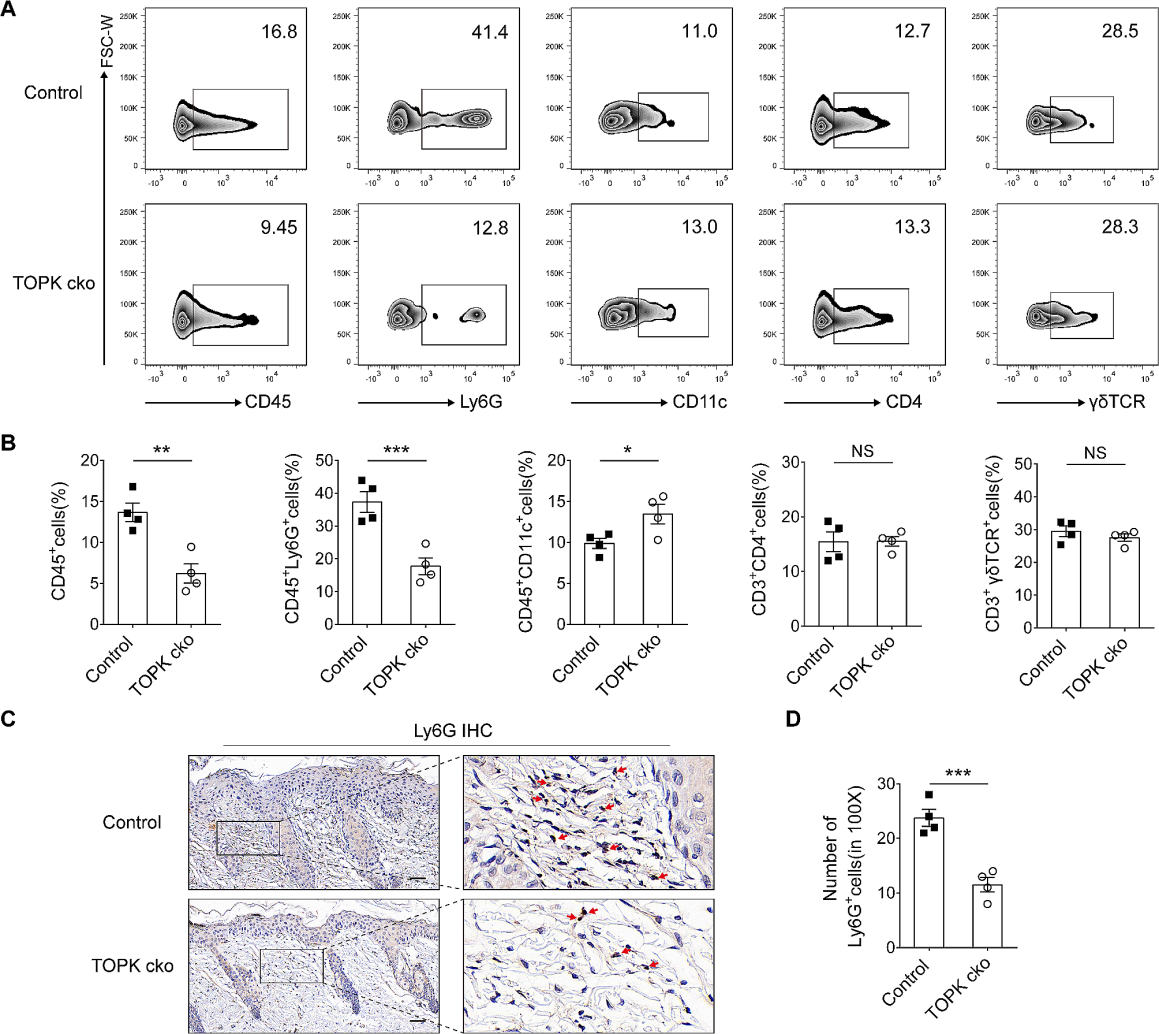
TOPK cko decreases neutrophils infiltration in the lesional skin of psoriatic model mice. (**A**) Flow cytometry analysis of representative immune cells in the lesional skin of control psoriatic model mice and TOPK cko psoriatic model mice. (**B**) Statistical analysis of the percentage of CD45^+^ cells, CD45^+^Ly6G^+^ cells, CD45^+^CD11c^+^ cells, CD3^+^CD4^+^ cells, and CD3^+^γδTCR^+^ cells in the lesional skin of control psoriatic model mice and TOPK cko psoriatic model mice. (**C**) Representative IHC staining images of anti-Ly6G antibody in lesional skin of control psoriatic model mice and TOPK cko psoriatic model mice. (**D**) Quantification analysis of CD45^+^Ly6G^+^ cells in the lesional skin in 100× magnification images. Scale bar = 50 μm. Data are representative of two independent experiments. Significance was determined by (B and D) Student’s t-test. *P < 0.05, **P < 0.01, ***P < 0.001

Previous studies have indicated that immune cells, including T cells, γδT cells, dendritic cells, and neutrophils, are involved in the pathogenesis of psoriasis [30]. In order to clarify the potential cellular mechanism involved, we next collected the lesional skin samples and performed flow cytometry analysis. The gating strategy is seen in supplementary material 3. The data indicated that the percentage of CD45^+^ immune cells infiltrated in the lesional skin was markedly decreased in TOPK cko psoriatic model mice. Specifically, the percentage of CD45^+^Ly6G^+^ cells was significantly decreased in the lesional skin of TOPK cko psoriatic model mice. The percentage of CD45^+^CD11c^+^ cells was slightly increased in the lesional skin of TOPK cko psoriatic model mice, which may be due to the decreased proportion of CD45^+^Ly6G^+^ cells. The percentage of CD3^+^CD4^+^ cells and CD3^+^γδTCR^+^ cells did not show any significant difference between control mice and TOPK cko mice (Fig. 3A and B). To further confirm the alteration of neutrophils, skin samples were subjected to IHC staining of anti-Ly6G antibody. The quantification results showed that the number of Ly6G^+^ cells in the dermis was significantly decreased in TOPK cko psoriatic model mice (Fig. 3C and D). Altogether, these results demonstrate that keratinocytes-expressed TOPK regulates neutrophils infiltration in psoriasis-like dermatitis.

### TOPK-regulated genes enrich IL-17 signaling pathway and regulate neutrophils chemotaxis in keratinocytes

**Fig. 4:**
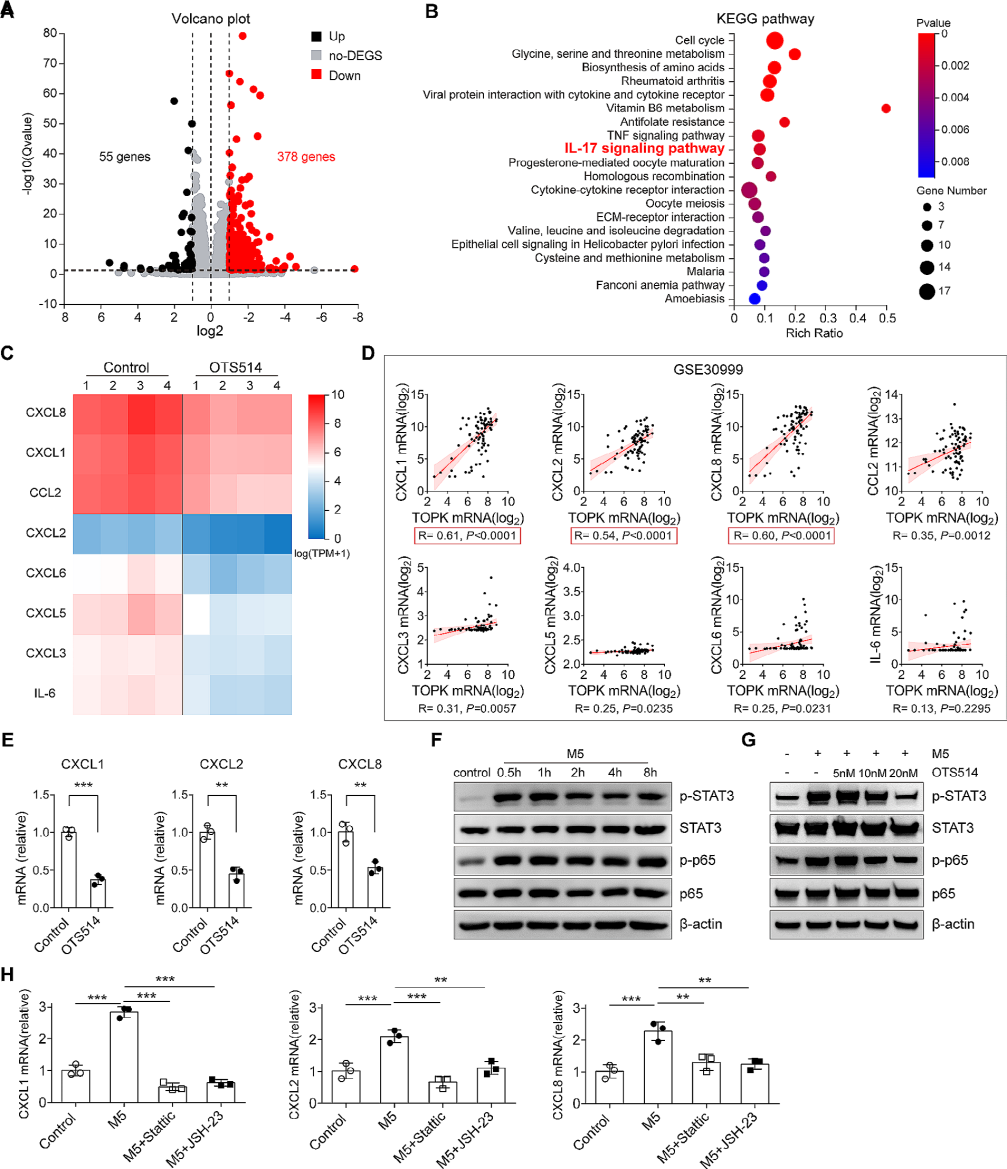
TOPK regulates the expression of neutrophils chemokines via activation of STAT3 and NF-κB p65 in keratinocytes. (**A**) Volcano plot of the upregulated and downregulated genes after OTS514 treatment (10nM) in HaCaT cells under M5 stimulation. (**B**) KEGG pathway enrichment analysis of the downregulated genes in HaCaT cells under M5 stimulation. (**C**) Heat map of the downregulated genes that enriched IL-17 signaling pathway. (**D**) Correlation analysis of TOPK and these downregulated genes (CXCL1, CXCL2, CXCL8, CCL2, CXCL3, CXCL5, CXCL6, and IL-6). The red marks represent the three genes with higher correlation. (**E**) RT-PCR analysis of neutrophils chemokines genes CXCL1, CXCL2, and CXCL8. (**F**) Western blot of total and phosphorylation of NF-κB p65 and STAT3 in HaCaT cells treated with M5 cytokines for the indicated time points. (**G**) Western blot of total and phosphorylation of NF-κB p65 and STAT3. HaCaT cells were preincubated with OTS514 for 6 h and then treated with M5 for 1 h with the indicated dose. (**H**) RT-PCR analysis of CXCL1, CXCL2, and CXCL8. HaCaT cells were preincubated with JSH-23 (30µM) and stattic (10µM) for 6 h and then treated with or without M5 cytokines for 24 h. Data are representative of two independent experiments. Significance was determined by (D) Student’s t-test and (H) one-way ANOVA followed by Dunnett’s multiple comparisons test. **P < 0.01, ***P < 0.001

To investigate the molecular mechanism underlying decreased neutrophils infiltration, we next examined the gene expression profiles by RNA sequencing to clarify the downstream targets of TOPK. HaCaT keratinocytes were cultured in M5 cocktail cytokines stimulation, treated with or without TOPK inhibitor OTS514 for 24 h, and then subjected to RNA sequencing. In all the differentially expressed genes, 55 genes were upregulated and 378 genes were downregulated (Fig. 4A). Enrichment analysis indicated that the downregulated genes enriched IL-17 signaling pathway (Fig. 4B), which is one of the most crucial signaling pathways in psoriasis pathogenesis and progression. Among these genes that enriched IL-17 signaling pathway, there were several neutrophils chemokines, including CXCL1, CXCL2, CXCL3, CXCL5, CXCL6, and CXCL8 (Fig. 4C). To further confirm the potential regulatory relationship between TOPK and these neutrophils chemokines, a GEO dataset containing multiple psoriatic samples was searched and analyzed. Correlation analysis indicated that among those neutrophils chemokines, CXCL1, CXCL2, and CXCL8 were highly positively correlated with the levels of TOPK in psoriatic lesions, further supporting that TOPK may regulate the expression of these three neutrophils chemokines (Fig. 4D). Subsequently, we performed RT-PCR to verify the regulatory effect of TOPK on the expression of CXCL1, CXCL2, and CXCL8. The results showed that TOPK inhibition significantly decreased the transcription levels of CXCL1, CXCL2, and CXCL8 (Fig. 4E). Collectively, the above data suggest that inhibiting TOPK in keratinocytes downregulates neutrophils chemokines expression, which may be the reason why specific deletion of TOPK attenuates psoriasis-like dermatitis in mice.

### TOPK inhibition suppresses the expression of neutrophils chemokines via inhibiting STAT3 and NF-κB p65 in keratinocytes

STAT3 and NF-κB p65 are two key transcription factors (TFs) in the pathogenesis of psoriasis, as well as two known downstream TFs of TOPK in cancer [31–33]. Therefore, we subsequently examined the activation of STAT3 and NF-κB p65 in keratinocytes in psoriasis-like conditions. Western blotting showed that phospho-STAT3 (p-STAT3) and phospho-NF-κB p65 (p-p65) were significantly increased after M5 cocktail cytokines treatment in as early as 0.5 h, and the activation of STAT3 and NF-κB p65 was maintained for more than 8 h (Fig. 4F). We then examined the effect of TOPK inhibition on the activation of STAT3 and NF-κB p65. The data indicated that TOPK inhibition obviously suppressed the upregulation of p-STAT3 and p-p65 induced by M5 cocktail cytokines, and the suppression efficiency was OTS514 dose-dependent (Fig. 4G). Afterwards, HaCaT cells were treated with stattic and JSH-23 to inhibit the activation of STAT3 and NF-κB p65 in vitro. RT-PCR results showed that inhibition of either STAT3 or NF-κB p65 significantly reduced the transcription levels of these three neutrophils chemokines, indicating that STAT3 and NF-κB p65 activation were necessary for the upregulation of CXCL1, CXCL2, and CXCL8 in psoriatic inflammatory environment (Fig. 4H). In summary, these data suggest that TOPK promotes the expression of neutrophils chemokines via regulating STAT3 and NF-κB p65 activation in keratinocytes.

### Neutrophils deletion diminishes the suppressive role of TOPK cko in psoriasis-like dermatitis in mice

**Fig. 5:**
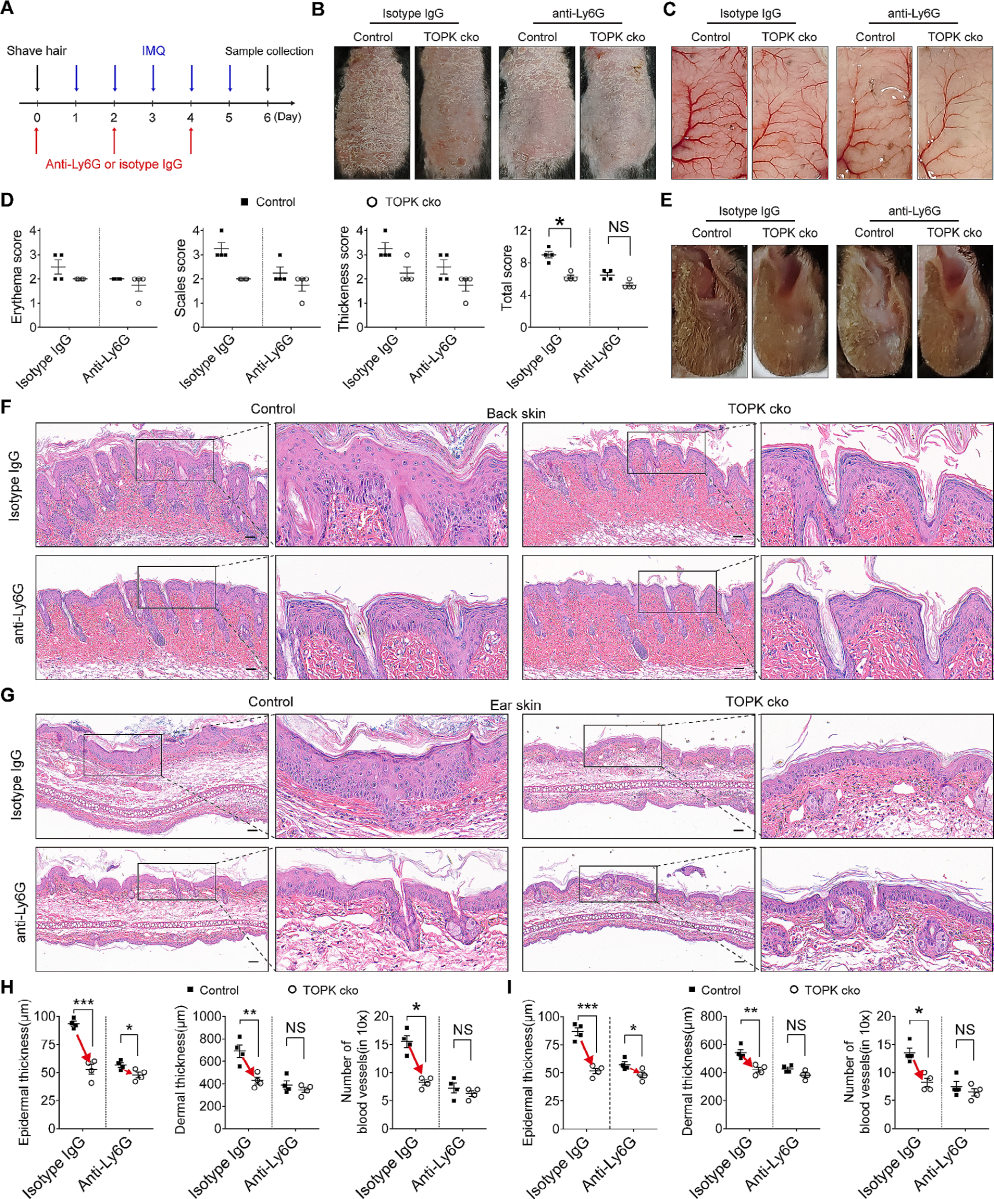
Neutrophils are required for the attenuated psoriasis-like dermatitis in TOPK cko mice. (**A**) Schematic of the treatment performed on mice. (**B**) Macroscopic images of psoriasis-like manifestations on the back skin of control psoriatic model mice and TOPK cko psoriatic model mice treated with isotype IgG or anti-Ly6G antibody. (**C**) Images of blood vessels within skin of control psoriatic model mice and TOPK cko psoriatic model mice treated with isotype IgG or anti-Ly6G antibody. (**D**) PASI scoring of the lesional skin in control psoriatic model mice and TOPK cko psoriatic model mice. The scores were assessed on day 6. *n* = 4 per group. (**E**) Macroscopic images of psoriasis-like manifestations on the ear skin of control psoriatic model mice and TOPK cko psoriatic model mice treated with isotype IgG or anti-Ly6G antibody. (**F** and **G**) Representative HE staining images of back (**F**) and ear (**G**) skin of control psoriatic model mice and TOPK cko psoriatic model mice treated with isotype IgG or anti-Ly6G antibody. (**H** and **I**) Statistical data of mice back skin (H) and ear skin (I) in epidermal thickness, dermal thickness and number of blood vessels. *n* = 4 per group. Scale bar = 100 μm. Significance was determined by (D) Mann-Whitney U test and (H and I) Student’s t-test. *P < 0.05, **P < 0.01, ***P < 0.001

To further determine the relationship between the suppressive role of TOPK cko in psoriasis and the decreased neutrophils infiltration, neutrophils were depleted by using the anti-Ly6G neutralizing antibody. These mice were then subjected to inducing psoriasis-like dermatitis. The schematic diagram of the experimental procedure is shown in Fig. 5A. The macroscopic images showed that, compared with WT mice, deletion of TOPK in keratinocytes markedly alleviated psoriasis-like manifestations, including scaling, erythema, and epidermal thickening, whereas after neutrophils depletion, psoriasis-like manifestations became similar between control mice and TOPK cko mice (Fig. 5B). In addition, blood vessel hyperplasia was obviously attenuated in TOPK cko psoriasis model mice, whereas after anti-Ly6G antibody treatment, blood vessel hyperplasia was no longer significantly different between control mice and TOPK cko mice (Fig. 5C). Similarly, PASI scoring showed that neutrophils depletion almost abrogated the decreased scores caused by TOPK conditional knockout (Fig. 5D). Psoriasis-like dermatitis was also induced by using mice ear skin. The macroscopic images of the ear showed that in control IgG-treated group, TOPK cko markedly attenuated psoriasis-like manifestations, while in neutrophils depletion group, TOPK specific deletion brought only a mild alleviation of the psoriasis-like manifestations (Fig. 5E). Notably, the same phenomenon was observed in histopathological changes. HE staining showed that no matter in back skin or in ear skin, the epidermal thickness, dermal thickness, and the number of blood vessels were significantly reduced in TOPK cko mice in isotype IgG treated group, whereas in neutrophils depletion group, the epidermal thickness was only slightly decreased in TOPK cko mice, and the dermal thickness and blood vessel numbers showed no statistical difference between control mice and TOPK cko mice (Fig. 5F-I). Taken together, these data indicate that the attenuated psoriasis-like dermatitis in TOPK cko mice is almost abrogated by neutrophils deletion, suggesting that TOPK promotes psoriatic progression largely by regulating neutrophils infiltration.

### TOPK inhibitor OTS154 attenuates psoriasis-like dermatitis in already-established psoriatic model mice

**Fig. 6:**
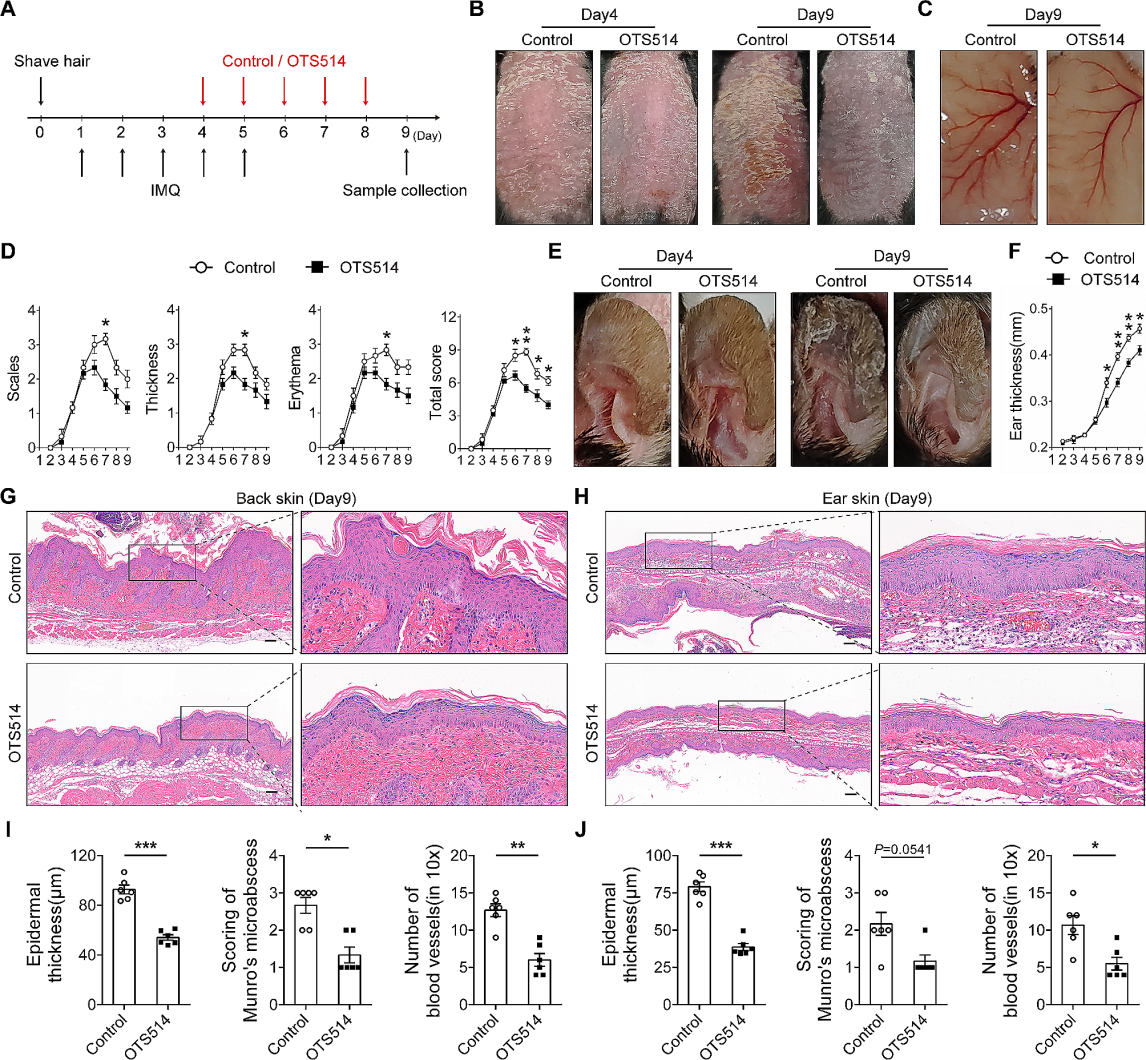
TOPK inhibitor OTS514 alleviates psoriasis-like dermatitis in already-established psoriatic model mice. (**A**) Schematic of the treatment performed on mice. (**B**) Macroscopic images (day 4 and day 9) of psoriasis-like manifestations on the back skin. The images of day 4 were taken before the cream treatment. (**C**) Images of the blood vessels (day 9) within the lesional back skin of control cream and OTS514 cream treated psoriatic model mice. (**D**) PASI scoring of the back lesional skin of control cream and OTS514 cream treated psoriatic model mice. The scores were assessed continuously from day 2 to day 9. *n* = 6 per group. (**E**) Macroscopic images of psoriasis-like manifestations on the ears of mice. (**F**) Statistical analysis of ear thickness measured by vernier caliper. *n* = 6 per group. (**G** and **H**) Representative HE staining images of mice back skin and ear skin treated with control cream and OTS514 cream. (**I** and **J**) Statistical data of mice back skin (**I**) and ear skin (**J**) in epidermal thickness, Munro’s microabscess scores and number of blood vessels. *n* = 6 per group. Scale bar = 100 μm. Data are representative of two independent experiments. Significance of Fig. 6D was determined by Mann-Whitney U test. Significance of Fig. 6F, I and J was determined by Student’s t-test or Mann-Whitney U test, as appropriate. *P < 0.05, **P < 0.01, ***P < 0.001

Next, TOPK inhibitor OTS514 was topically applied to explore the potential therapeutic efficacy of targeting TOPK in psoriasis. OTS514 was manufactured into the cream formulation and then topically smeared on the lesional skin of already-established (IMQ pre-treated for three days) psoriatic model mice. The schematic diagram of the experimental procedure is shown in Fig. 6A. The results indicated that the psoriasis-like manifestations, including erythema, scaling, and epidermal thickening, showed no obvious difference between the two groups at the baseline (day 4), whereas the disease manifestations were markedly alleviated after five days of OTS514 cream treatment (Fig. 6B). Vessel hyperplasia of the lesional skin was also ameliorated after OTS514 cream treatment for five days (Fig. 6C). PASI scoring indicated that OTS514 cream treatment attenuated the disease progression and promoted the remission of psoriasis-like dermatitis (Fig. 6D). In addition to the back skin, similar results were obtained on the ear skin. The disease manifestations of psoriasis-like dermatitis in mice ear skin were obviously alleviated after smearing OTS514 cream (Fig. 6E). Vernier caliper measuring indicated that the ear thickness of OTS514 cream treated mice was significantly thicker than that of control cream treated mice in IMQ-induced psoriatic model (Fig. 6F). Pathologically, HE staining indicated that topical application of OTS514 attenuated psoriasis-like pathological changes, including epidermal thickening, Munro’s microabscess, and blood vessel hyperplasia in both back and ear skin (Fig. 6G-J). Altogether, these results demonstrate that topically applying TOPK inhibitor attenuates psoriatic progression and promotes disease remission in psoriasis-like dermatitis in mice.

## Discussion

Apart from the known oncogenic role of TOPK, this study identified that TOPK is also implicated in the pathogenesis of the inflammatory disease psoriasis. Previous studies have identified TOPK as a crucial oncogene. In malignant diseases, TOPK has been found to be strongly expressed in various tumors, including melanoma, breast cancer, colorectal cancer, lung cancer, liver cancer, prostate cancer, renal cancer, leukemia, and lymphoma [22]. TOPK could phosphorylate a number of downstream factors, such as STAT3, NF-κB, ERK2, PRPK, c-jun, histone H2AX, and histone H3, thereby activating downstream signaling pathways [19]. Inhibition of TOPK with inhibitors has been reported to potently inhibit the proliferation of multiple cancers [34–37]. In addition, clinical research recently documented that TOPK expression was highly related to the poor outcomes of cancer patients [38–40]. TOPK, as a promising therapeutic target of anti-cancer, has aroused extensive attention of basic researchers and clinicians. Although a recent study reported that the compound worenine attenuated psoriasis inflammation in psoriatic model mice by inhibiting TOPK activity and that TOPK may affect IL-17 signaling pathway in phosphoproteomic analysis [26], the detailed mechanism by which TOPK contributes to psoriasis progression still needs further investigation. In the present study, by using multiple experimental approaches, we identified a pivotal role of keratinocyte-expressed TOPK in psoriatic inflammation and uncovered the mechanism involved. TOPK upregulates in psoriatic keratinocytes, and keratinocytes-expressing TOPK contributes to the pathogenesis of psoriasis-like dermatitis by promoting neutrophils recruitment in the lesional skin. However, as psoriatic pathogenesis, progression, maintenance, and relapse are of great complexity, TOPK roles in different stages of psoriasis still deserve further investigation.

Multiple TOPK inhibitors have been developed, and targeting TOPK may be a potential therapeutic option for the treatment of psoriasis. Since TOPK has been considered to be a promising target for suppressing tumor malignant proliferation, small-molecule compounds targeting TOPK are increasingly being developed. Among all the inhibitors, OTS514, OTS964, and HI-TOPK-032 are currently the most widely used TOPK inhibitors [37, 41]. OTS964 is a derivative of OTS514, and both of them have high selectivity and affinity for TOPK [37]. Targeting TOPK with OTS514 or OTS964 has been shown to effectively inhibit the growth of many human cancer cells [42], such as lung cancer and colorectal cancer [36, 43], and to suppress the metastasis of ovarian cancer [35, 44]. HI-TOPK-032 belongs to the class of ATP competitive inhibitors. Computer modeling indicated that HI-TOPK-032 binds with the active site of TOPK, and studies reported that HI-TOPK-032 effectively inhibits TOPK both in vitro and in vivo [41, 45]. These previous studies strongly support that targeting TOPK by specific inhibitors is a potential therapeutic approach in cancer. Apart from the above three inhibitors, our previous study reported a novel TOPK inhibitor named paeonol. Paeonol could effectively target TOPK and inhibit downstream p38 MAPK and JNK signaling pathways, thereby suppressing solar UV-induced skin dermatitis [24]. In a recent study, we preliminarily found that TOPK increased in psoriasis lesions and pre-application of OTS514 inhibited the pathogenesis of psoriasis-like dermatitis in mice [25]. In the present study, our data showed that TOPK was mainly increased in epidermal keratinocytes and post-application of TOPK inhibitor OTS514 significantly attenuated psoriasis-like manifestations and pathological changes in already-established psoriatic model mice. Therefore, given these potent TOPK inhibitors and our findings, we propose that targeting TOPK may be a promising therapeutic option for psoriasis treatment. However, due to the wide variety of TOPK inhibitors, further studies on the efficacy and safety of TOPK inhibitors are needed.

Although TOPK has been reported to regulate a variety of signaling pathways in cancer, the role of TOPK in modulating the IL-17 signaling pathway has not been identified. The IL-17 signaling pathway has been extensively studied and identified as one of the most important signaling pathways in psoriasis, while the regulatory factor modulating IL-17 signaling pathway in psoriasis has not been fully elucidated. In this study, we examined the relationship between IL-17A and TOPK in skin lesions of psoriasis patients and found that TOPK levels were positively correlated with IL-17A levels. Besides, RNA-seq revealed that inhibiting TOPK repressed the transcription of some genes in IL-17 signaling pathway. Furthermore, inhibiting TOPK downstream TFs suppressed the transcription of some genes associated with neutrophils chemotaxis in IL-17 signaling pathway. Our study indicated that TOPK regulates downstream genes of IL-17 signaling pathway to modulate neutrophils infiltration in psoriasis, suggesting that TOPK may be a modulator of IL-17 signaling pathway in psoriatic dermatitis. In summary, our study identifies that TOPK regulates the downstream genes of IL-17 signaling pathway to promote neutrophils infiltration in psoriasis.

Keratinocytes-expressed TOPK facilitates psoriasis progression mainly by promoting the recruitment of neutrophils. Previous studies have identified that neutrophils play critical roles in psoriasis pathogenesis and progression. Shao and colleagues reported that activating neutrophils aggregated psoriasis-like manifestations in mice model [17]. It was also found that the ratio of neutrophils vs. lymphocytes was elevated in psoriasis patients [46], and the ratio was closely associated with the severity of psoriasis [47]. Kristian and colleagues found that clinical use of anti-IL-17A antibodies probably targeted neutrophils-released IL-17A to disrupt keratinocytes-neutrophils communication, thereby alleviating psoriatic skin inflammation [48]. Studies reported that neutrophils promote the pathogenesis and development of psoriasis through a number of approaches, which include forming Munro’s microabscess [49], generating neutrophil extracellular traps [50], producing inflammatory cytokines and proinflammatory factors [16, 51], and accelerating innate immune response [52]. Depletion of neutrophils by the neutralizing antibody was found to significantly attenuate the development of psoriasis dermatitis in a murine model of psoriasis [53]. Importantly, in our study, TOPK cko reduced neutrophils infiltration and attenuated psoriasis-like dermatitis in mice. Although biological deletion of neutrophils did not completely abolish the effect of TOPK cko, it greatly diminished the attenuated role of TOPK cko, suggesting that the pro-inflammatory role of TOPK in psoriasis is largely mediated by facilitating neutrophils infiltration. Keratinocytes-expressed TOPK regulated the expression of chemokines to promote neutrophils infiltration, thereby exacerbating psoriasis progression. However, RNA-seq showed that except for neutrophils chemokines, there were several other genes regulated by TOPK, which may also have somewhat promotive effects in the progression of psoriasis.

## Conclusions

Our study identified a pivotal role of the oncogenic kinase TOPK in contributing to psoriasis progression. Increased expression of TOPK in keratinocytes promoted the progression of psoriasis via regulating neutrophils infiltration. Topical application of TOPK inhibitor alleviated disease progression in already-established psoriasis-like dermatitis in mice. This study highlights the role of keratinocytes-expressed TOPK in psoriasis and proposes that topically targeting TOPK as a potential therapeutic approach for psoriasis treatment.

### Electronic supplementary material

Below is the link to the electronic supplementary material.

Supplementary Material 1

Supplementary Material 2

Supplementary Material 3

Supplementary Material 4

## Data Availability

Data availability statementThe datasets generated during the current study have been deposited in the GEO datasets at: https://www.ncbi.nlm.nih.gov/geo/query/acc.cgi? acc=GSE253203.

## Abbreviations

TOPK: T-LAK cell-oriented protein kinase
GEO: Gene Expression Omnibus
GSE: Gene Expression Omnibus Series
IHC: Immunohistochemical
RT-RCR: Real-time polymerase chain reaction
CXCL: C-X-C motif chemokine ligand
STAT3: Signal transducer and activator of transcription 3
NF-κB: Nuclear factor kappa-B
AMPs: Antimicrobial peptides
LCN2: Lipocalin-2
MAPKK: Mitogen-activated protein kinase kinase
HUST: Huazhong University of Science and Technology
IL: Interleukin
TNF: Tumor necrosis factor
IMQ: Imiquimod
PASI: Psoriasis area and severity index
