# Supplementary material for: The oncogenic kinase TOPK upregulates in psoriatic keratinocytes and contributes to psoriasis progression by regulating neutrophils infiltration
Source: Cell Commun Signal. 2024 Aug 1;22:386. doi: 10.1186/s12964-024-01758-9 (PMC11292866; doi:10.1186/s12964-024-01758-9)
Supplement: Supplementary file 1 — Supplementary Material 1 [file 12964_2024_1758_MOESM1_ESM.pdf]

Supplementary figure 1

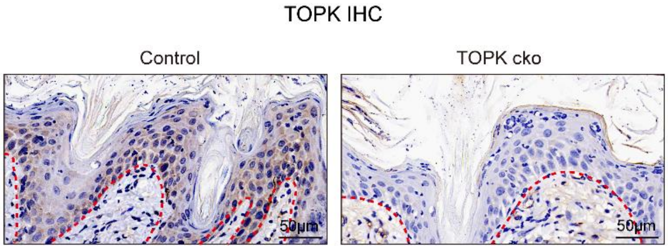

Figure s1. IHC staining of anti-TOPK antibody using the back skin of control psoriatic model mice and TOPK cko psoriatic model mice. Scale bar = 50 μm.
