# Supplementary material for: The oncogenic kinase TOPK upregulates in psoriatic keratinocytes and contributes to psoriasis progression by regulating neutrophils infiltration
Source: Cell Commun Signal. 2024 Aug 1;22:386. doi: 10.1186/s12964-024-01758-9 (PMC11292866; doi:10.1186/s12964-024-01758-9)
Supplement: Supplementary file 2 — Supplementary Material 2 [file 12964_2024_1758_MOESM2_ESM.pdf]

Supplementary figure 2

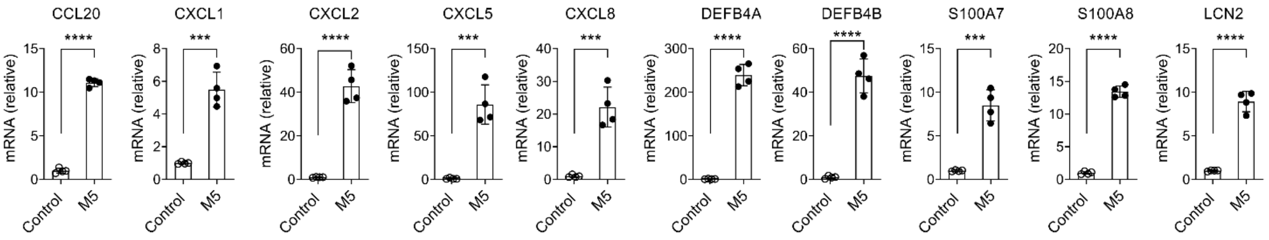

Figure s2. M5 cocktail cytokines induce the upregulation of psoriasis related T cell chemokines, neutrophil chemokines, and antimicrobial peptides in HaCat cells.
