# Supplementary figures and images for: The oncogenic kinase TOPK upregulates in psoriatic keratinocytes and contributes to psoriasis progression by regulating neutrophils infiltration
Source: Cell Commun Signal. 2024 Aug 1;22:386. doi: 10.1186/s12964-024-01758-9 (PMC11292866; doi:10.1186/s12964-024-01758-9)

### Supplementary figure 3

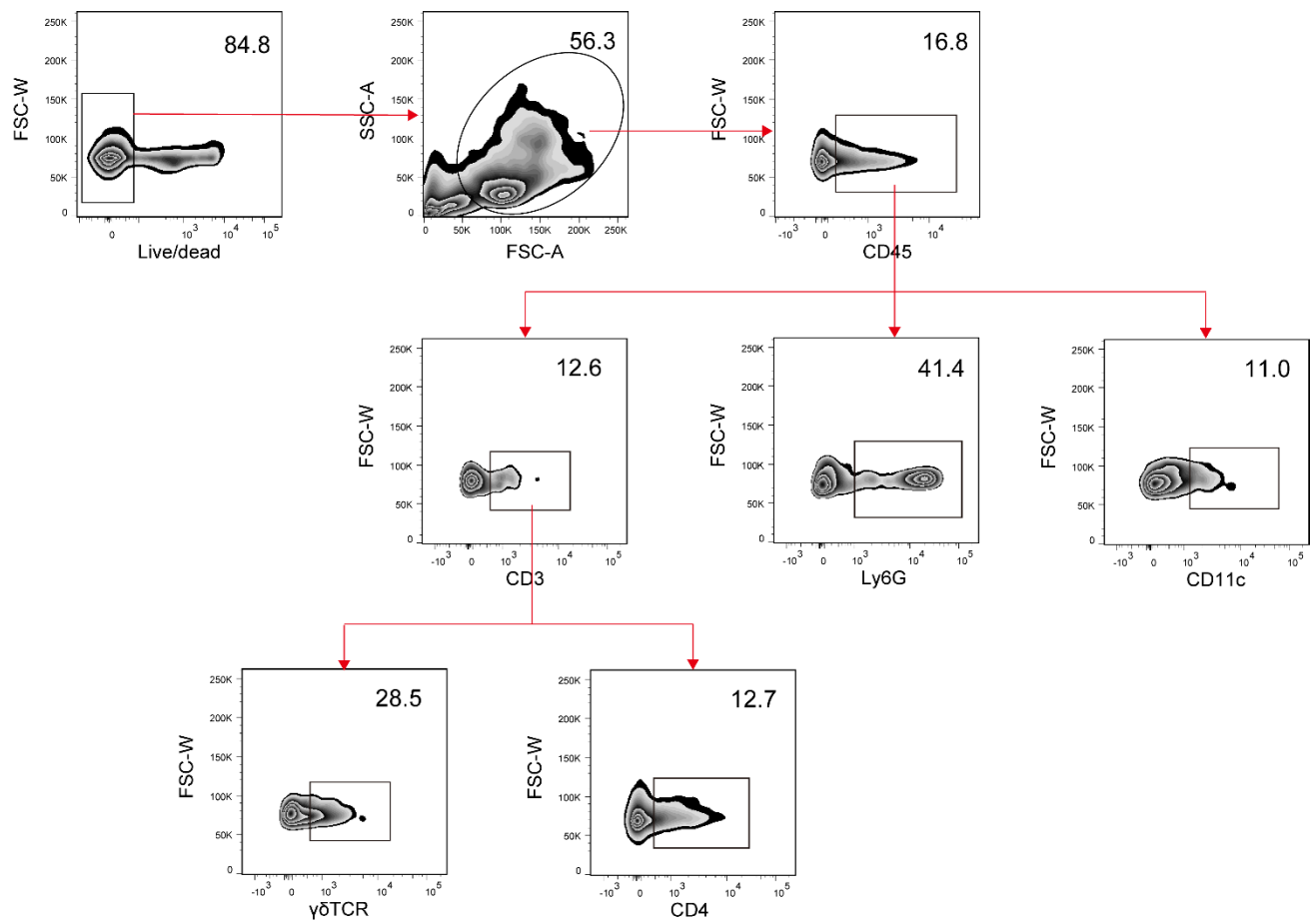

Figure s3. Gating strategy of flow cytometry in this study.

Supplement: Supplementary file 3 — Supplementary Material 3 [file 12964_2024_1758_MOESM3_ESM.pdf]
