# Supplementary material for: The oncogenic kinase TOPK upregulates in psoriatic keratinocytes and contributes to psoriasis progression by regulating neutrophils infiltration
Source: Cell Commun Signal. 2024 Aug 1;22:386. doi: 10.1186/s12964-024-01758-9 (PMC11292866; doi:10.1186/s12964-024-01758-9)
Supplement: Supplementary file 4 — Supplementary Material 4 [file 12964_2024_1758_MOESM4_ESM.pdf]

Supplemental materials and methods

GEO data analysis

The public datasets used for analysis in this study were searched from the National Center for Biotechnology Information (www.ncbi.nlm.nih.gov/geoprofiles) with the keywords “psoriasis” and selecting the organism “Homo sapiens” or “Mus musculus”. Then, the appropriate GEO datasets were downloaded and analyzed.

Preparation of OTS514 cream

OTS514 hydrochloride was ordered from Selleck (cat: S7652). The OTS514 cream and control cream were prepared as previously reported [1]. The cream used in this study contains 0.5 mg/mL OTS514 hydrochloride.

Scoring of psoriasis-like dermatitis severity

The severity of psoriasis-like dermatitis in psoriatic model mice was determined using the PASI scoring. Briefly, the lesional skin of each mouse was scored independently for erythema, scaling and epidermal thickness. According to the PASI, the scale ranges from 0 to 4. 0 means none, 1 means mild, 2 means moderate, 3 means severe, and 4 means very severe. The cumulative score was used as an overall assessment of psoriasis severity (on a scale of 0-12). For assessing the severity of Munro's microabscesses, Munro's microabscesses were scored from 0 to 4. 0 means none, 1 means mild, 2 means moderate, 3 means marked, and 4 means very marked.

Hematoxylin-eosin (HE) staining and immunohistochemical (IHC) staining

The HE staining and IHC staining were conducted following the standard operating procedures as previously reported [2]. Mice skin samples were embedded in paraffin

after being fixed with 4% paraformaldehyde at room temperature for 24 hours. Anti-TOPK antibody (cat: 4942T, Cell Signaling Technology (CST); cat: sc-293028, Santa Cruz) was used for the IHC staining.

Histological analysis

The Pannoramic MIDI (3DHISTECH Ltd., Hungary) was used to scan the HE and IHC stained sections under the same parameters. For histological analysis, epidermal thickness, dermal thickness, scoring of Munro's microabscesses, and quantifying dermal blood vessels were analyzed using CaseViewer software (3DHISTECH Ltd., Hungary). Blood vessels were quantified in the images of 10× magnification. The intensity of IHC staining in epidermal keratinocytes, as previously described, was assessed by integrated optical density (IOD)/area by using Image-Pro Plus software (Media Cybernetics, USA) [3].

Depletion of Neutrophils

Neutrophils were depleted, as previously reported [4-6]. For depletion of neutrophils, 100 ug Ultra-LEAF™ purified anti-mouse Ly-6G (Biolegend, cat: 127632) were intraperitoneally injected for three times as indicated. The control group received the same dose of control isotype IgG (bio-legend, cat:400544). Neutrophil depletion was periodically evaluated by differential counting by collecting blood samples from the caudal vein.

Real-time PCR (RT-PCR) analysis

The total RNA of HaCaT cells was extracted using TRIzol reagent (Invitrogen) and then reverse-transcribed into cDNA with the Reverse Transcription Kit (Thermo Fisher). RT-

PCR was performed using CFX96 (Bio-Rad). Primers were synthesized by Sangong Biotechnology Co., Ltd. (Shanghai, China). The results are shown as relative levels normalized to β-actin. The primers are as follows:

cxcl1, F: CCAAGAACATCCAAAGTGTGAAC,

R: TTTGTCACTGTTCAGCATCTTTTC;

cxcl2, F: CACGCTCTCCGCCGC,

R: CACCTTCACACTTTGGATGTTCTT;

cxcl8, F: ATACTCCAAACCTTTCCACCC,

R: AACTTCTCCACAACCCTCTGC;

β-actin, F: TAGTTGCGTTACACCCTTTCTTG,

R: TCACCTTCACCGTTCCAGTTT.

Reference

[1] F. Zeng, H. Lu, M. Wu, C. Dai, J. Li, J. Wang, G. Hu, Topical application of TOPK inhibitor OTS514 suppresses psoriatic progression by inducing keratinocytes cell cycle arrest and apoptosis, Experimental dermatology, 32 (2023) 1823-1833.

[2] F. Zeng, H. Chen, L. Chen, J. Mao, S. Cai, Y. Xiao, J. Li, J. Shi, B. Li, Y. Xu, Z. Tan, F. Gong, B. Li, Y. Qian, L. Dong, F. Zheng, An Autocrine Circuit of IL-33 in Keratinocytes Is Involved in the Progression of Psoriasis, Journal of Investigative Dermatology, 141 (2021) 596-606.e597.

[3] X.L. Jia, S.Y. Li, S.S. Dang, Y.A. Cheng, X. Zhang, W.J. Wang, C.E. Hughes, B. Caterson, Increased expression of chondroitin sulphate proteoglycans in rat hepatocellular carcinoma tissues, World journal of gastroenterology, 18 (2012) 3962-3976.

[4] J.M. Daley, A.A. Thomay, M.D. Connolly, J.S. Reichner, J.E. Albina, Use of Ly6G-specific monoclonal antibody to deplete neutrophils in mice, J Leukoc Biol, 83 (2008) 64-70.

[5] D. Sun, P. Sun, S. He, M. Shi, Rat IgG mediated circulatory cell depletion in mice requires mononuclear phagocyte system and is facilitated by complement, J Leukoc Biol, 107 (2020) 529-539.

[6] G. Han, A. Havnaer, H.H. Lee, D.J. Carmichael, L.R. Martinez, Biological depletion of neutrophils attenuates pro-inflammatory markers and the development of the psoriatic phenotype in a murine model of psoriasis, Clin Immunol, 210 (2020) 108294.
